# Tree diversity drives diversity of arthropod herbivores, but successional stage mediates detritivores

**DOI:** 10.1002/ece3.3411

**Published:** 2017-09-18

**Authors:** Michael J. O'Brien, Matteo Brezzi, Andreas Schuldt, Jia‐Yong Zhang, Keping Ma, Bernhard Schmid, Pascal A. Niklaus

**Affiliations:** ^1^ Estación Experimental de Zonas Áridas Consejo Superior de Investigaciones Científicas Almería Spain; ^2^ Department of Evolutionary Biology and Environmental Studies University of Zurich Zurich Switzerland; ^3^ Institute of Global Health University of Geneva Geneva Switzerland; ^4^ German Centre for Integrative Biodiversity Research (iDiv) Halle‐Jena‐Leipzig Leipzig Germany; ^5^ Institute of Ecology Zhejiang Normal University Jinhua Zhejiang Province China; ^6^ State Key Laboratory of Vegetation and Environmental Change Institute of Botany Chinese Academy of Sciences Beijing China

**Keywords:** BEF‐China, biodiversity, canopy layers, community composition, ecosystem functioning, forest succession, plant–herbivore interactions, trophic groups

## Abstract

The high tree diversity of subtropical forests is linked to the biodiversity of other trophic levels. Disentangling the effects of tree species richness and composition, forest age, and stand structure on higher trophic levels in a forest landscape is important for understanding the factors that promote biodiversity and ecosystem functioning. Using a plot network spanning gradients of tree diversity and secondary succession in subtropical forest, we tested the effects of tree community characteristics (species richness and composition) and forest succession (stand age) on arthropod community characteristics (morphotype diversity, abundance and composition) of four arthropod functional groups. We posit that these gradients differentially affect the arthropod functional groups, which mediates the diversity, composition, and abundance of arthropods in subtropical forests. We found that herbivore richness was positively related to tree species richness. Furthermore, the composition of herbivore communities was associated with tree species composition. In contrast, detritivore richness and composition was associated with stand age instead of tree diversity. Predator and pollinator richness and abundance were not strongly related to either gradient, although positive trends with tree species richness were found for predators. The weaker effect of tree diversity on predators suggests a cascading diversity effect from trees to herbivores to predators. Our results suggest that arthropod diversity in a subtropical forest reflects the net outcome of complex interactions among variables associated with tree diversity and stand age. Despite this complexity, there are clear linkages between the overall richness and composition of tree and arthropod communities, in particular herbivores, demonstrating that these trophic levels directly impact each other.

## 1 INTRODUCTION

Plant diversity is important for maintaining ecosystem functioning and for supporting the diversity of other trophic levels (Balvanera et al., 2006; Hooper et al., 2005; Isbell et al., 2015; Siemann, Tilman, Haarstad, & Ritchie, 1998). Associations between the diversity of plants and other trophic levels have been studied intensively in grasslands (Haddad et al., 2009; Scherber et al., 2010; Siemann et al., 1998), but the relationship between plant and animal diversity in forests has received far less attention. Most research examining the effect of plant diversity on animal communities in forests has focused on the loss of tree species due to land‐use change and logging (Dunn, 2004; Edwards et al., 2010; Ewers et al., 2015; Fredericksen & Fredericksen, 2004; Tews et al., 2004). However, understanding the variables that drive the distribution, diversity and abundance of trophic levels that depend upon forest vegetation in a contiguous forest landscape can provide insights into the factors that promote biodiversity and ecosystem functioning in subtropical forests.

An important relationship in terrestrial ecosystems is that between plants and arthropods because of the feedbacks that exist between these groups of organisms. Plants provide habitat and food while arthropods may alter plant diversity (Bagchi et al., 2014; Have et al., 2006; Kempel et al., 2015), contribute to decomposition (Donoso, Johnston, Clay, & Kaspari, 2013), mediate plant reproduction (Gonzalez‐Megias, 2016; Zvereva, Lanta, & Kozlov, 2010), and disperse seeds (Kalisz, Hanzawa, Tonsor, Thiede, & Voigt, 1999). Relationships between plant and arthropod diversity may depend on the dominant plant life form in the ecosystem—i.e., forests dominated by trees and grasslands dominated by herbaceous plants may have different plant–arthropod interactions.

Forests have physical attributes for arthropod communities that are different from grasslands because of their structural complexity, which may supersede the effects of plant diversity on arthropod diversity (Southwood, Brown, & Reader, 1979). Forests have high spatial heterogeneity with horizontal variation (gap dynamics) in stem densities, light and temperature (Chazdon & Fetcher, 1984; Chen et al., 1999; Raich, 1989), and vertical variation in quality and quantity of leaf and woody tissues (Ellsworth & Reich, 1993). Vertical canopy strata can have direct effects on arthropod communities. For example, birds and other predators in the canopy may reduce abundances of pollinators and herbivores (Van Bael, Brawn, & Robinson, 2003) and variation in light and microclimate can change the nutrients in leaf tissues (Le Corff & Marquis, 1999). Variation in abiotic conditions such as soil characteristics and climatic variables associated with altitude and topography (Paoli, 2006; Paoli, Curran, & Zak, 2006; Pendry & Proctor, 1997; Proctor, Lee, Langley, Munro, & Nelson, 1988) may also influence arthropod communities independent of tree diversity. In addition, trees produce recalcitrant tissues that decompose slowly and create biotope space that persists for long times. For example, fallen or standing dead plant material provides space for breeding and larval development regardless of the surrounding living tree species (Irmler, Heller, & Warning, 1996; Jacobs, Spence, & Langor, 2007; Schiegg, 2000). Combined, these variables make forests distinct from grasslands, which may alter plant–arthropod relationships.

Furthermore, the long‐lived nature of trees means that a forest consists of a mosaic of stand age classes due to disturbances that occur at different spatial and temporal scales (Bergeron, 2000). Large canopy gaps promote the recruitment of early‐successional tree species with traits for establishment and rapid growth in high light environments while undisturbed areas will have long‐lived species with traits that support shade tolerance and stress resistance (Iida et al., 2014; Kohyama, Suzuki, Partomihardjo, Yamada, & Kubo, 2003). Environmental conditions in older forest stands will have decreased light and soil drying and understories will be cooler than younger forest stands in gaps (Chen et al., 1999; Raich, 1989). Therefore, stand age, which correlates with functional traits, environmental conditions, and the quantity of woody debris (Chen et al., 1999; Jacobs et al., 2007; Raich, 1989), may mediate arthropod diversity across the landscape more than tree species diversity.

These factors of stand age, heterogeneity in spatial structure and environmental conditions, may supersede the effects of tree diversity on arthropod diversity in forests. However, the strength and magnitude of the tree–arthropod diversity relationship may also differ between different arthropod functional groups occupying different trophic niches. For example, detritivores will respond to food quantity and quality that may be less related to living tree diversity than to species identity or functional traits of dead plant material that effect detritus quantity and quality (Donoso et al., 2013; Graça, Pozo, Canhoto, & Elosegi, 2002; Hansen, 2000; Hättenschwiler & Jørgensen, 2010). In contrast, herbivore richness and abundance should be more directly linked to living tree diversity because herbivores feed on these plants (Andow, 1991; Knops et al., 1999). Predators, in turn, may be indirectly linked to plants through alterations in the diversity and abundance of herbivores (Hutchinson, 1959; Knops et al., 1999) and may therefore show a weaker tree–arthropod diversity relationship (Balvanera et al., 2006). Pollinators may operate independently of stand‐level tree diversity altogether due to their dependence on flowering and potentially long‐distance travel (Bawa, Bullock, Perry, Coville, & Grayum, 1985; Sobek, Tscharntke, Scherber, Schiele, & Steffan‐Dewenter, 2009). Therefore, to determine the factors that mediate arthropod distributions across a forested landscape, arthropod functional groups must be assessed separately while accounting for tree diversity and forest age.

In this study, we tested the effect of forest variables (e.g., tree species diversity, stand age, and vertical position) and arthropod community characteristics (e.g., taxon richness and abundance) using a comparative study design across gradients of tree diversity and stand age in a subtropical forest. We selected forest plots so that these gradients were relatively independent, allowing us to separate the effects of tree diversity and stand age (Baruffol et al., 2013; Bruelheide et al., 2011). We sampled arthropods of four functional groups (i.e., detritivores, herbivores, pollinators, and predators) in the understorey and the canopy to assess differences in vertical position as well. We posit that these variables will differentially affect the different arthropod functional groups. We further assessed whether arthropod community compositions were related to tree community composition. Given the host specificity of some arthropods, we hypothesized that the species compositions of tree and arthropod communities should show different associations depending on the arthropod functional group considered (as outlined above).

## 2 MATERIALS AND METHODS

### 2.1 Experimental design

In June 2010, we placed arthropod traps in 27 plots of a comparative study in the Gutianshan National Nature Reserve (GNNR) in Zhejiang Province, China (Baruffol et al., 2013; Bruelheide et al., 2011; Castro‐Izaguirre et al., 2016). The plots in the GNNR were established in 2008 to encompass a gradient of stand ages (20–120 years) and tree species richness levels (25–69 species of all trees greater than 10 cm diameter at breast height). Four to seven plots were assigned to five strata based on successional stage of the forests stands (<20 years; <40 years; <60 years; <80 years; >80 years). Within each stratum, plots spanned a gradient of lower to higher tree species diversity (Table S1). Bruelheide et al. (2011) presented the age and diversity spread of these plots, indicating their continuous distribution (see Figure 4 in Bruelheide et al. (2011) see Table S1). The forest is continuous (see Fig. S1) and classified as subtropical with ~2,000 mm of rain mainly falling from March to September (Yu, Hu, Yu, Ding, & Fang, 2001). The 27 plots are 30 × 30 m in size, and their altitude ranges from 250 to 900 m above sea level. The average distance among plots was 3,400 m (95% CI: 535–7420).

Plots were subdivided into nine 10 × 10 m subplots. In July and August 2010, ten yellow sticky traps (9 × 11 cm sticky area, double face, MIOPLANT, Switzerland) were placed in each study plot. Five traps were suspended by bamboo sticks, in the understorey, 2 m above ground with one trap placed in the middle subplot and one in each of the four corner subplots (at least 5 m from the plot edge). The sticky sides of the understorey traps faced east‐west. Five additional traps were placed in the canopy, suspended from trees. One tree from each of the five most abundant species in the plot was chosen at random to suspend a trap in the canopy. Tree traps were positioned inside the middle of the crown of each tree between 3 and 18 m above the ground (tree heights ranged from 7 to 30 m) hanging from 0.35 mm fishing line and stabilized by small ballasts. Due to logistic constraints, trap exposure varied from 6 to 9 days depending on the plot. After collection, the traps were covered with plastic and stored in a freezer until further processing. Because yellow sticky traps present a sampling bias, absolute values of arthropod abundance cannot be estimated by our study. However, their use across all plots allows comparison of abundances among plots and should not inhibit the total number of species captured (Hoback, Svatos, Spomer, & Higley, 1999; Missa et al., 2009).

### 2.2 Arthropod sorting and counting

Arthropods were identified directly on the traps and were classified by order and to morpho‐species based on external morphological characteristics (Yuan, Zhang, Feng, & Hua, 2006; Zheng & Gui, 1999). Larvae were considered as separate morpho‐species because their diet often differs from their respective adult form and the difficulty in defining larvae to the correct adult morpho‐species. However, the ambiguity in larvae identification did not affect our results as larvae only represented 0.4% of the arthropods captured (123 individuals). Based on the inspection of the arthropod mouthparts, taxonomic experience, and known arthropod populations in the province, each morpho‐species was assigned to one of six functional groups: (1) herbivore folivores, (2) herbivore sapsuckers, (3) predators (including parasitoids), (4) detritivores, (5) pollinators (nonherbivorous), and (6) miscellaneous arthropods that could not be classified more precisely. The use of mouth parts was employed for insects that could not be assigned to a family containing only one functional type. These initial groups were aggregated into four classes for analysis: (1) herbivores (folivores + sapsuckers), (2) predators, (3) pollinators, and (4) detritivores. Insects with ambiguous classification were set to miscellaneous and are not included in our analysis (17% of the total). In total, we collected 28,198 arthropods belonging to 17 different orders and 598 morpho‐species (Table S2 and S3). Some morpho‐species may have been wrongly assigned to a functional group because these assignments were based on taxonomy and morphology (of mouth parts), and direct observations of feeding behavior were not made (Table S4). Therefore, sensitivity analysis was performed whereby the orders Lepidoptera, Hemiptera, and Coleoptera were either removed or all designated as herbivores. This analysis showed that the results were not dependent upon the feeding group assignment of these orders (Table S5).

### 2.3 Data analysis

We performed two sets of analyses: The first tested the effect of tree richness and stand age on arthropod richness and abundance, and the second assessed evidence for relationships between tree and arthropod compositions.

In order to assess the importance of tree diversity and stand age on arthropod diversity and abundance, we modeled arthropod richness and abundance, separately for each functional group, as a function of stand age (a continuous variable), tree species richness (a continuous variable, trees with a diameter at breast height of at least 10 cm), vertical position (a fixed factor with two levels; understorey and canopy), and all two‐way interactions between the continuous variables and vertical position using a linear mixed‐effects model. We also included a covariate for altitude. Because of low arthropod abundance on some traps, we aggregated traps from the same vertical position within plots which combined to 54 observations (2 sampling heights × 27 plots). To meet assumptions of linearity, arthropod richness and abundance were log‐transformed after adding one to account for zeros. Plot (a factor with 27 levels) and trap exposure time (a factor with 22 levels) were fit as random terms.

To test for specific associations between arthropod and tree composition, we first calculated the dissimilarities in tree community composition among plots using the Jaccard index based on tree species presence–absence per plot (Fig. S2). We performed the same analysis on the arthropod community composition among plots for each functional group separately. These dissimilarity matrices were transformed into two‐dimensional space by principal coordinate analysis (PCoA), and the first two axes were used as orthogonal metrics of differences in tree or arthropod community composition among plots. The Bray–Curtis index (with square‐root transformed abundances) was also calculated for arthropod functional groups, but results were similar to those using the Jaccard index (Fig. S3–S6). Therefore, only results from analysis of the Jaccard data are discussed.

We performed constrained analysis of proximities on the Jaccard distance matrix of the arthropod community for each functional group to test the effect of stand age and tree composition on arthropod community composition. The PCoA ordination of the communities of each functional group was constrained by the two PCoA axes derived from the PCoA of tree composition, stand age, and tree species richness. We tested the significance of the constraining terms with a permutation test. If the inertia in the permuted models was lower than in the constrained model, then the association modeled was considered statistically significant.

We further tested the importance of tree composition for arthropod composition of each functional group using the PCoA axis 1 of tree composition as a predictor in a linear model with type‐I sum of squares of each PCoA axis of the arthropod functional group compositions. Therefore, if tree composition affected the arthropod community composition of a functional group, then the PCoA axis of tree composition would significantly correlate with the first or second PCoA axis of the arthropod functional group. In other words, plots with a more similar tree community composition would have more similar arthropod community composition. Furthermore, we tested for the independence of tree composition from stand age by adding stand age in before tree composition, and if tree composition was independent of stand age, then its importance would be retained even after fitting stand age first in the model.

All linear and mixed models were performed with the asreml‐R package (ASReml 3, VSN International, Hemel Hempstead, UK), using R 3.3.2 (http://r-project.org). The vegdist function in vegan package (Oksanen et al., 2015) was used to calculate Jaccard dissimilarities. Principal coordinate ordination was carried out with the cmdscale function, and the constrained analysis of proximities was performed with the capscale function (Legendre & Anderson, 1999) in the vegan package.

## 3 RESULTS

**Figure 1:**
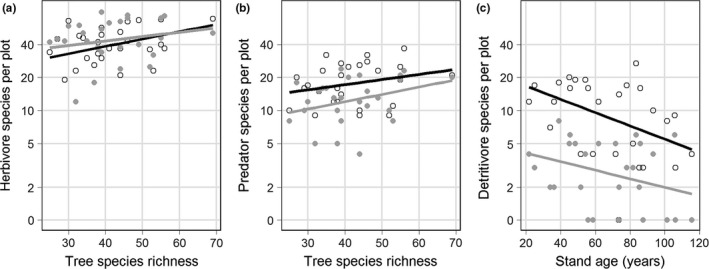
The effect of tree species richness and stand age on arthropod richness of different functional groups. (a) The relationship between herbivore richness and tree species richness for both understorey (black lines and points) and canopy (gray lines and points) traps. (b) The relationship between predator richness and tree species richness for understorey and canopy traps. (c) The relationship between detritivore richness and stand age in the understory and canopy traps. Arthropod richness was log‐transformed but presented with axes back‐transformed.

**Figure 2:**
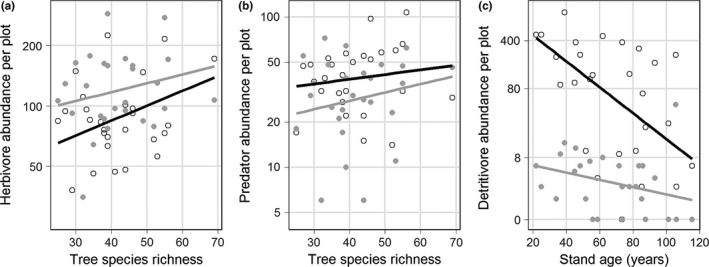
The effect of tree species richness and stand age on arthropod abundance of different functional groups. (a) Herbivore abundance increased with species richness in the canopy (gray lines and points) and understorey (black lines and points). (b) Predator abundance increased in the understorey and canopy traps but was always higher in the understorey. (c) Detritivore abundance declined with stand age but faster in understorey traps. Arthropod abundance was log‐transformed but presented with axes back‐transformed.

Arthropod richness and abundance were differentially affected by tree species richness, stand age, and vertical position, depending on the arthropod functional group (Figures 1 and 2; Tables S7 and S8). Herbivore richness significantly increased with tree species richness (Figure 1a) while herbivore abundance was significantly higher in the canopy than in the understorey (Figure 2a). In addition, stand age had a significant negative effect on herbivore richness despite a tendency for later successional stages to have higher tree species richness. Predator richness and abundance were significantly higher in the understorey than in the canopy (Figures 1b and 2b) and increased nonsignificantly with tree species richness. Detritivore richness and abundance significantly decreased with stand age and were significantly higher in the understory than in the canopy (Figures 1c and 2c). Detritivores were unaffected by tree species richness. In addition, detritivore abundance decreased significantly faster in the understorey than in the canopy as stand age increased (Figure 2c). Pollinator richness and abundance were not affected by any variables and were generally found in equal numbers in all plots. ANOVA tables for all richness and abundance analyses are in Tables S7 and S8.

**Figure 3:**
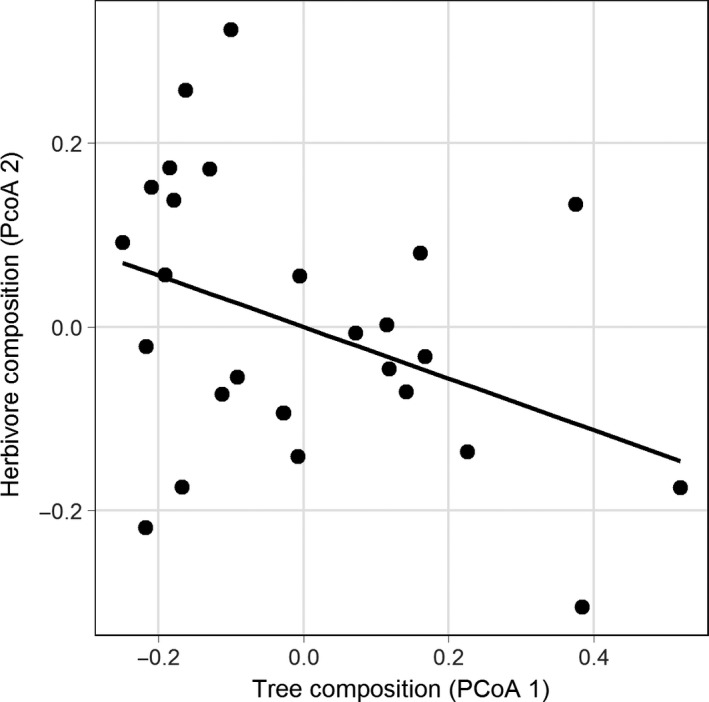
Tree composition correlated with herbivore community composition. A relationship between principal coordinates of tree and herbivore community composition was found. Plots with more similar tree communities had more similar herbivore communities.

The constrained analysis of proximities on the different arthropod communities indicated that only compositions of herbivore and detritivore groups were explained by tree species composition (Figs S3–S6), as evidenced by the PCoA axes of tree composition explaining herbivore composition (*p* < .1) and detritivore composition (*p* < .1). In our direct comparison of PCoA axis of tree composition to the PCoA axes of each arthropod functional group (Figs S7–S10), only herbivore composition was related to tree composition. The PCoA axis 2 of herbivore community composition was related to the tree community composition (*F*_1,25_ = 4.4, *p* < .05 and *F*_1,25_ = 2.5, *p* = .1 for tree composition fit before and after stand age, respectively; Figure 3). However, the community compositions of the other arthropod functional groups were not related to tree composition regardless of the inclusion of stand age in the model.

## 4 DISCUSSION

In this assessment of arthropod communities across a gradient of tree species richness and stand age, we found that different arthropod functional groups showed different associations with the forest characteristics. Specifically, herbivore richness and abundance increased with tree species richness, and detritivore richness and abundance decreased with forest age. In contrast, the richness and abundance of predators and pollinators were not strongly associated with tree diversity or stand age. Therefore, higher tree diversity only sustained a higher diversity of herbivores, but stand age and vertical position superseded the effects of tree diversity on detritivores. Furthermore, a direct link between herbivore community composition and tree community composition was found, which suggests a potential role for species‐specific interactions between herbivores and trees in this subtropical forest.

### 4.1 Tree and herbivore diversity

Herbivores showed a clear relationship with tree species richness. Most likely the connection between species‐rich tree communities and more diverse herbivore communities was due to more tree species supporting a greater array of feeding demands combined with the benefits provided by feeding on a diversity of plants that improves overall diet and fitness (Coley & Barone, 1996). Recent work by Brezzi, Schmid, Niklaus, and Schuldt (2017) showed higher levels of feeding on locally rare species, indicating that generalists or at least nonspecialists had a strategy to increase their diversity of food intake. Although only marginally significant, herbivore abundance also increased with tree diversity, in support of growing evidence that herbivory increases with tree species richness (Brezzi et al., 2017; Scherber et al., 2006; Schuldt et al., 2010; Vehviläinen, Koricheva, & Ruohomäki, 2007).

Herbivore community composition was also linked to tree community composition. Whether these results support the role of herbivores in promoting a diversity–productivity relationship depends on the feeding preferences of these herbivores (Barone, 1998). If host‐specific feeding dominates or if host‐specific feeders concentrate in low‐diversity forest stands and generalists are more common in high‐diversity stands, then herbivores would inhibit productivity of low‐diversity forests and promote a biodiversity effect (Barone, 1998; Root, 1973; Schuldt et al., 2010; Zhang et al., 2017). Furthermore, recent research suggests that more diverse forest stands may have increased nutrient cycling rates due to faster leaf turnover, a pattern that may be mediated by higher herbivore diversity (Huang et al., 2017). Although our results cannot determine feeding preferences or underlying biodiversity mechanisms, they clearly show greater tree diversity sustains a more diverse herbivore community.

### 4.2 Stand age and detritivore associations

Detritivore richness and abundance were negatively related to stand age. Early‐successional forest stands have canopies dominated by fast growing light‐demanding trees with slow‐growing, shade‐tolerant species recruiting underneath. Fast growing trees have higher leaf nutrient content and less recalcitrant foliage (Eichenberg, Trogisch, Huang, He, & Bruelheide, 2013; Garnier et al., 2004; Li, Pei, Kéry, Niklaus, & Schmid, 2017), and these characteristics promote higher quality detritus which would support greater diversity and abundance of detritivores (Cortez, Garnier, Pérez‐Harguindeguy, Debussche, & Gillon, 2007). In addition, the understorey supported greater richness and abundance, which is likely due to the greater quantity of dead and down tissue than the canopy. The lack of evidence for a relationship between tree species diversity and detritivores and the marginal evidence for a role of tree composition supports our initial hypotheses that stand age can supersede the importance of tree species diversity.

### 4.3 What shapes predator and pollinators communities?

Predator richness and abundance also tended to increase with tree species richness, although the relationship was statistically not significant. The weak response of predators to tree species richness may indicate an indirect effect mediated by the herbivore community (Balvanera et al., 2006; Knops et al., 1999). The pattern of herbivores and predators tracking the next lower trophic level (i.e., predators follow herbivores follow plants) supports the diversity–trophic structure hypothesis (Hutchinson, 1959; Knops et al., 1999; Murdoch, Evans, & Peterson, 1972). The weakening of the positive trend of predators with trees suggests a bottom‐up effect in support of Balvanera et al. (2006). A positive signal with only 27 plots suggests that biologically tree diversity is showing important cascading effects on arthropod functional groups.

Pollinators in general were operating independent of any forest characteristics. We suspect pollinator beta‐diversity is driven not by tree species diversity, but instead specifically by flowering diversity and phenology. Therefore, without data on the diversity of flowering species, we are unable to determine mechanisms promoting this functional group.

## 5 CONCLUSION

In the subtropical forest studied here, more diverse tree communities hosted more diverse and abundant herbivore communities (and to a weaker extent predator communities), a pattern which was not found in other arthropod guilds. Tree and herbivore community compositions were also correlated, indicating a degree of specialized herbivory. Therefore, tree diversity drives herbivore diversity in spite of variables associated with stand age and spatial heterogeneity. In contrast, detritivores were responding to stand age which likely represents a proxy for functional traits associated with different tree growth strategies. Combined, these results indicate the importance of maintaining tree species and functional diversity across forest successional stages for promote arthropod diversity in subtropical forests.

## CONFLICT OF INTEREST

None declared.

## AUTHOR CONTRIBUTIONS

MOB analyzed the data, wrote the manuscript, and led the revisions. MB conceived and carried out the experiment and contributed to revisions. AS provided conceptual development with expertise on arthropods and contributed to revisions. JYZ contributed to experimental design and insect identification. KM provided logistical support for working in China and the setup of the larger experiment. BS and PAN wrote the grant that funded the project and contributed to the experimental design, analysis, writing, and revisions.
