# Supplementary material for: Tree diversity drives diversity of arthropod herbivores, but successional stage mediates detritivores
Source: Ecol Evol. 2017 Sep 18;7(21):8753–60. doi: 10.1002/ece3.3411 (PMC5677472; doi:10.1002/ece3.3411)
Supplement: Supplementary file 1 [file ECE3-7-8753-s001.docx]

**Supporting information**

**Table S1. Plot descriptions**

**Table S2. Summary of morpho-species abundance**

**Table S3. Summary of abundance by functional group**

**Table S4. Attached Excel file of arthropod identification**

**Table S5. ANOVA table for sensitivity analysis for richness.**

**Table S6. ANOVA table for sensitivity analysis for abundance.**

**Table S7. ANOVA table for species richness.**

**Table S8. ANOVA table for abundance.**

**Fig. S1. Map of the plot distribution.**

**Fig. S2. PCoA for tree composition.**

**Fig. S3 – S6. Distance-based redundancy analysis for each functional group.**

**Fig. S7 – S10. PCoA of each functional group with both Jaccard and Bray-Curtis.**

**Tree diversity drives diversity of arthropod herbivores but successional stage mediates detritivores**

Michael J. O’Brien^1,2^***, Matteo Brezzi^2,3^, Andreas Schuldt^4^, Jia-Yong Zhang^5^, Keping Ma^6^, Bernhard Schmid^2^ & Pascal A. Niklaus^2*^

*^1^Estación Experimental de Zonas Áridas, Consejo Superior de Investigaciones Científicas, Carretera de Sacramento s/n, E-04120 La Cañada, Almería, Spain*

*^2^Department of Evolutionary Biology and Environmental Studies, University of Zurich, Switzerland*

*^3^Institute of Global Health, University of Geneva, 9 Chemin des Mines, 1202 Geneva*

*^4^German Centre for Integrative Biodiversity Research (iDiv), Halle-Jena-Leipzig*

*Deutscher Platz 5e, 04103 Leipzig, Germany*

*^5^Institute of Ecology, Zhejiang Normal University, Jinhua, Zhejiang Province, China*

*^6^State Key Laboratory of Vegetation and Environmental Change, Institute of Botany, Chinese*

*Academy of Sciences, Beijing 100093, China*

**corresponding author email:* pascal.niklaus@ieu.uzh.ch and mikey.j.obrien@gmail.com

**Table S1.** Description of plots.

| **Plot** | **Elevation** | **Northing** | **Easting** | **Tree count** | **Tree species richness** | **Stand age (y)** | **Detritivore**  **count** | **Folivore**  **count** | **Sap sucker count** | **Predator count** | **Pollinator count** |
| --- | --- | --- | --- | --- | --- | --- | --- | --- | --- | --- | --- |
| 1 | 522 | 29.23957 | 118.11587 | 353 | 44 | 72 | 13 | 2 | 167 | 19 | 807 |
| 2 | 590 | 29.24926 | 118.13484 | 476 | 69 | 74 | 381 | 26 | 251 | 73 | 867 |
| 3 | 720 | 29.23885 | 118.12402 | 949 | 49 | 54 | 115 | 10 | 219 | 104 | 411 |
| 4 | 542 | 29.24963 | 118.12015 | 296 | 44 | 88 | 21 | 2 | 148 | 48 | 516 |
| 5 | 507 | 29.24687 | 118.1122 | 207 | 25 | 85 | 3 | 7 | 181 | 33 | 367 |
| 6 | 880 | 29.25497 | 118.14747 | 656 | 39 | 56 | 125 | 6 | 214 | 94 | 204 |
| 7 | 903 | 29.25184 | 118.14373 | 740 | 46 | 78 | 132 | 5 | 238 | 77 | 585 |
| 8 | 413 | 29.24106 | 118.11019 | 490 | 53 | 73 | 8 | 2 | 182 | 69 | 264 |
| 9 | 348 | 29.24275 | 118.11337 | 383 | 55 | 84 | 310 | 5 | 485 | 111 | 572 |
| 10 | 670 | 29.25188 | 118.15791 | 358 | 41 | 86 | 72 | 19 | 123 | 78 | 217 |
| 11 | 647 | 29.27383 | 118.13647 | 646 | 35 | 62 | 474 | 14 | 94 | 71 | 216 |
| 12 | 620 | 29.24939 | 118.1219 | 404 | 29 | 106 | 2 | 9 | 119 | 76 | 368 |
| 13 | 402 | 29.2463 | 118.11621 | 245 | 32 | 59 | 3 | 2 | 142 | 36 | 204 |
| 14 | 639 | 29.24944 | 118.13518 | 385 | 38 | 116 | 5 | 9 | 228 | 63 | 284 |
| 15 | 618 | 29.24917 | 118.13106 | 309 | 39 | 82 | 11 | 9 | 136 | 30 | 256 |
| 16 | 309 | 29.24253 | 118.09966 | 1195 | 37 | 52 | 10 | 0 | 170 | 50 | 211 |
| 17 | 310 | 29.24342 | 118.10828 | 665 | 39 | 45 | 101 | 5 | 506 | 94 | 513 |
| 18 | 569 | 29.24516 | 118.12461 | 543 | 52 | 101 | 24 | 17 | 128 | 35 | 465 |
| 19 | 655 | 29.28045 | 118.08389 | 1233 | 30 | 25 | 492 | 15 | 296 | 71 | 525 |
| 20 | 679 | 29.28139 | 118.08803 | 947 | 38 | 36 | 98 | 12 | 145 | 42 | 406 |
| 21 | 566 | 29.27059 | 118.08084 | 537 | 56 | 106 | 295 | 12 | 237 | 167 | 272 |
| 22 | 617 | 29.27698 | 118.09066 | 1135 | 33 | 22 | 492 | 5 | 215 | 109 | 439 |
| 23 | 419 | 29.2145 | 118.13723 | 776 | 55 | 34 | 232 | 14 | 154 | 66 | 216 |
| 24 | 366 | 29.21483 | 118.13469 | 462 | 34 | 48 | 252 | 5 | 256 | 99 | 199 |
| 25 | 345 | 29.21713 | 118.13155 | 513 | 27 | 45 | 387 | 2 | 219 | 100 | 291 |
| 26 | 251 | 29.21489 | 118.12155 | 803 | 44 | 39 | 1044 | 8 | 263 | 95 | 106 |
| 27 | 665 | 29.24709 | 118.13605 | 414 | 46 | 93 | 192 | 8 | 246 | 134 | 206 |

**Table S2.** The average abundance per plot of each order for understorey and canopy positions, and the total number of morpho-species of every order.

| **Order** | **Postion** | **Mean abundance (se)** | **Number of morpho-species** | **Total**  **abundance** |
| --- | --- | --- | --- | --- |
| Acari | Understorey  Canopy | 5.8 (2.2)  1.7 (0.3) | 12 | 148 |
| Araneae | Understorey  Canopy | 10.3 (1.8)  2.5 (0.4) | 64 | 347 |
| Blattodea | Understorey  Canopy | 0  1 | 1 | 2 |
| Coleoptera | Understorey  Canopy | 7.3 (0.9)  6.8 (1.1) | 42 | 380 |
| Collembola | Understorey  Canopy | 193 (46.1)  3.5 (1.7) | 21 | 5122 |
| Dermaptera | Understorey  Canopy | 1  0 | 1 | 1 |
| Diptera | Understorey  Canopy | 212.9 (25.1)  250.7 (25.4) | 99 | 12515 |
| Ephemeroptera | Understorey  Canopy | 0  1 | 1 | 2 |
| Hemiptera | Understorey  Canopy | 90.3 (9.0)  123.2 (11.4) | 209 | 5764 |
| Hymenoptera | Understorey  Canopy | 47.1 (6.1)  37 (3.7) | 54 | 2272 |
| Isoptera | Understorey  Canopy | 0.6 (0.2)  1.4 (0.7) | 2 | 10 |
| Lepidoptera | Understorey  Canopy | 2.7 (0.3)  6.7 (1.2) | 33 | 253 |
| Mecoptera | Understorey  Canopy | 0  1 | 1 | 1 |
| Odonata | Understorey  Canopy | 0.3 (0.3)  0.3 (0.3) | 2 | 2 |
| Orthoptera | Understorey  Canopy | 3.0 (0.8)  4.2 (0.5) | 23 | 178 |
| Phasmatodea | Understorey  Canopy | 0.8 (0.3)  0.5 (0.2) | 5 | 8 |
| Thysanoptera | Understorey  Canopy | 20.2 (3.2)  29.5 (5.2) | 28 | 1193 |

**Table S3.** The average abundance per plot of each feeding guild for understorey and canopy positions.

| **Feeding guild** | **Position** | **Abundance (se)** | **Total abundance** | **Total richness** |
| --- | --- | --- | --- | --- |
| Detritivores | Understorey  Canopy | 198 (46)  5 (2) | 5286 | 38 |
| Folivores | Understorey  Canopy | 4 (1)  5 (1) | 230 | 43 |
| Miscellaneous | Understorey  Canopy | 72 (8)  110 (10) | 4889 | 65 |
| Pollinators | Understorey  Canopy | 183 (21)  187 (21) | 9987 | 150 |
| Predators | Understorey  Canopy | 43 (4)  33 (3) | 2044 | 94 |
| Sap-suckers | Understorey  Canopy | 90 (9)  123 (11) | 5762 | 208 |

**Table S5.** The ANOVA tables from the sensitivity analysis of (A) herbivore richness, (B) predator richness, (C) detritivore richness and (D) pollinator richness with the orders Lepidotera, Hemiptera and Coleoptera either removed or all set as herbivores.

| **Source of variation** | **d.f.** | **denominator d.f.** | **F_removed_** | **F_all_herbivores_** |
| --- | --- | --- | --- | --- |
| **A** |  |  |  |  |
| Altitude | 1 | 22.9 | 1.0 | 1.1 |
| Stand age | 1 | 23 | 6.9* | 5.1* |
| Tree richness | 1 | 22.9 | 6.1* | 6.0* |
| Trap position | 1 | 24 | 2.5 | 2.1 |
| Age X position | 1 | 24 | 3.0^†^ | 3.3^†^ |
| Richness X position | 1 | 24 | 0.9 | 0.7 |
| **Variance components** | **Var.** | **SE** |  |  |
| Exposure period | -0.01 | 0.04 |  |  |
| Plot | 0.10 | 0.06 |  |  |
| Variance understorey | 0.00 | 0.02 |  |  |
| Variance canopy | 0.11 | 0.04 |  |  |
| **B** |  |  |  |  |
| Altitude | 1 | 23 | 0.2 | 0.2 |
| Stand age | 1 | 23 | 0.4 | 0.4 |
| Tree richness | 1 | 21.9 | 2.3 | 2.3 |
| Trap position | 1 | 24 | 24.6*** | 24.6*** |
| Age X position | 1 | 24 | 0.0 | 0.0 |
| Richness X position | 1 | 24 | 0.4 | 0.4 |
| **Variance components** | **Var.** | **SE** |  |  |
| Exposure period | 0.01 | 0.08 |  |  |
| Plot | 0.13 | 0.09 |  |  |
| Variance understorey | 0.04 | 0.03 |  |  |
| Variance canopy | 0.09 | 0.03 |  |  |
| **C** |  |  |  |  |
| Altitude | 1 | 22.9 | 0.2 | 0.3 |
| Stand age | 1 | 22.9 | 3.6^†^ | 3.4^†^ |
| Tree richness | 1 | 23 | 0.8 | 0.8 |
| Trap position | 1 | 24 | 85.7*** | 83.4*** |
| Age X position | 1 | 24 | 0.7 | 0.5 |
| Richness X position | 1 | 24 | 0.5 | 0.4 |
| **Variance components** | **Var.** | **SE** |  |  |
| Exposure period | -0.05 | 0.19 |  |  |
| Plot | 0.38 | 0.24 |  |  |
| Variance understorey | 0.27 | 0.12 |  |  |
| Variance canopy | 0.16 | 0.11 |  |  |
| **D** |  |  |  |  |
| Altitude | 1 | 22.4 | 1.1 | 1.6 |
| Stand age | 1 | 23 | 0.1 | 0.1 |
| Tree richness | 1 | 14 | 2.5 | 2.9 |
| Trap position | 1 | 24 | 0.3 | 0.4 |
| Age X position | 1 | 24 | 0.4 | 0.2 |
| Richness X position | 1 | 24 | 0.5 | 1.1 |
| **Variance components** | **Var.** | **SE** |  |  |
| Exposure period | 0.03 | 0.03 |  |  |
| Plot | 0.01 | 0.02 |  |  |
| Variance understorey | 0.03 | 0.02 |  |  |
| Variance canopy | 0.04 | 0.02 |  |  |

d.f., degrees of freedom; denominator d.f., denominator degrees of freedom,

F, conditional F-statistic; Var., variance component estimate and SE, standard errors for random effects; ^†^P<0.1, *****P < 0.05, ******P < 0.01, *******P < 0.001

**Table S6.** The ANOVA tables from the sensitivity analysis of (A) herbivore abundance, (B) predator abundance, (C) detritivore abundance and (D) pollinator abundance with the orders Lepidotera, Hemiptera and Coleoptera either removed or all set as herbivores.

| **Source of variation** | **d.f.** | **denominator d.f.** | **F_removed_** | **F_all_herbivores_** |
| --- | --- | --- | --- | --- |
| **A** |  |  |  |  |
| Altitude | 1 | 22.8 | 0.9 | 0.9 |
| Stand age | 1 | 23 | 1.2 | 0.9 |
| Tree richness | 1 | 15.7 | 4.0^†^ | 4.2 |
| Trap position | 1 | 24 | 12.3** | 13.7** |
| Age X position | 1 | 24 | 2.4 | 2.2 |
| Richness X position | 1 | 24 | 0.6 | 0.6 |
| **Variance components** | **Var.** | **SE** |  |  |
| Exposure period | -0.05 | 0.04 |  |  |
| Plot | 0.13 | 0.07 |  |  |
| Residual variance | 0.11 | 0.03 |  |  |
| **B** |  |  |  |  |
| Altitude | 1 | 23 | 0.0 | 0.0 |
| Stand age | 1 | 23 | 0.3 | 0.3 |
| Tree richness | 1 | 21.4 | 1.0 | 1.0 |
| Trap position | 1 | 24 | 7.8* | 7.8* |
| Age X position | 1 | 24 | 1.8 | 1.8 |
| Richness X position | 1 | 24 | 0.2 | 0.2 |
| **Variance components** | **Var.** | **SE** |  |  |
| Exposure period | 0.02 | 0.15 |  |  |
| Plot | 0.17 | 0.16 |  |  |
| Residual variance | 0.18 | 0.05 |  |  |
| **C** |  |  |  |  |
| Altitude | 1 | 22.9 | 0.0 | 0.0 |
| Stand age | 1 | 23 | 6.4* | 6.4* |
| Tree richness | 1 | 22.5 | 2.9 | 2.9 |
| Trap position | 1 | 24 | 129.1*** | 129.1*** |
| Age X position | 1 | 24 | 7.3* | 7.3* |
| Richness X position | 1 | 24 | 3.4^†^ | 3.4^†^ |
| **Variance components** | **Var.** | **SE** |  |  |
| Exposure period | 0.00 | 0.62 |  |  |
| Plot | 1.03 | 0.76 |  |  |
| Residual variance | 0.86 | 0.25 |  |  |
| **D** |  |  |  |  |
| Altitude | 1 | 23 | 1.8 | 1.7 |
| Stand age | 1 | 23 | 0.1 | 0.1 |
| Tree richness | 1 | 21.7 | 1.8 | 1.8 |
| Trap position | 1 | 24 | 0.0 | 0.0 |
| Age X position | 1 | 24 | 0.3 | 0.3 |
| Richness X position | 1 | 24 | 0.5 | 0.4 |
| **Variance components** | **Var.** | **SE** |  |  |
| Exposure period | 0.02 | 0.12 |  |  |
| Plot | 0.15 | 0.13 |  |  |
| Residual variance | 0.13 | 0.04 |  |  |

d.f., degrees of freedom; denominator d.f., denominator degrees of freedom,

F, conditional F-statistic; Var., variance component estimate and SE, standard errors for random effects; ^†^P<0.1, *****P < 0.05, ******P < 0.01, *******P < 0.001

**Table S7.** The ANOVA tables from the linear mixed-effects model of (A) herbivore richness, (B) predator richness, (C) detritivore richness and (D) pollinator richness.

| **Source of variation** | **d.f.** | **denominator d.f.** | **F** |
| --- | --- | --- | --- |
| **A** |  |  |  |
| Altitude | 1 | 22.9 | 1.0 |
| Stand age | 1 | 23 | 6.9* |
| Tree richness | 1 | 22.9 | 6.1* |
| Trap position | 1 | 24 | 2.5 |
| Age X position | 1 | 24 | 3.0^†^ |
| Richness X position | 1 | 24 | 0.9 |
| **Variance components** | **Var.** | **SE** |  |
| Exposure period | -0.01 | 0.04 |  |
| Plot | 0.10 | 0.06 |  |
| Variance understorey | 0.00 | 0.02 |  |
| Variance canopy | 0.11 | 0.04 |  |
| **B** |  |  |  |
| Altitude | 1 | 23 | 0.2 |
| Stand age | 1 | 23 | 0.4 |
| Tree richness | 1 | 21.9 | 2.3 |
| Trap position | 1 | 24 | 24.6*** |
| Age X position | 1 | 24 | 0.0 |
| Richness X position | 1 | 24 | 0.4 |
| **Variance components** | **Var.** | **SE** |  |
| Exposure period | 0.01 | 0.08 |  |
| Plot | 0.13 | 0.09 |  |
| Variance understorey | 0.04 | 0.03 |  |
| Variance canopy | 0.09 | 0.03 |  |
| **C** |  |  |  |
| Altitude | 1 | 22.9 | 0.2 |
| Stand age | 1 | 22.9 | 3.6^†^ |
| Tree richness | 1 | 23 | 0.8 |
| Trap position | 1 | 24 | 85.7*** |
| Age X position | 1 | 24 | 0.7 |
| Richness X position | 1 | 24 | 0.5 |
| **Variance components** | **Var.** | **SE** |  |
| Exposure period | -0.05 | 0.19 |  |
| Plot | 0.38 | 0.24 |  |
| Variance understorey | 0.27 | 0.12 |  |
| Variance canopy | 0.16 | 0.11 |  |
| **D** |  |  |  |
| Altitude | 1 | 22.4 | 1.1 |
| Stand age | 1 | 23 | 0.1 |
| Tree richness | 1 | 14 | 2.5 |
| Trap position | 1 | 24 | 0.3 |
| Age X position | 1 | 24 | 0.4 |
| Richness X position | 1 | 24 | 0.5 |
| **Variance components** | **Var.** | **SE** |  |
| Exposure period | 0.03 | 0.03 |  |
| Plot | 0.01 | 0.02 |  |
| Variance understorey | 0.03 | 0.02 |  |
| Variance canopy | 0.04 | 0.02 |  |

d.f., degrees of freedom; denominator d.f., denominator degrees of freedom,

F, conditional F-statistic; Var., variance component estimate and SE, standard errors for random effects; ^†^P<0.1, *****P < 0.05, ******P < 0.01, *******P < 0.001

**Table S8.** The ANOVA tables from the linear mixed-effects model of (A) herbivore abundance, (B) predator abundance, (C) detritivore abundance and (D) pollinator abundance.

| **Source of variation** | **d.f.** | **denominator d.f.** | **F** |
| --- | --- | --- | --- |
| **A** |  |  |  |
| Altitude | 1 | 22.8 | 0.9 |
| Stand age | 1 | 23 | 1.2 |
| Tree richness | 1 | 15.7 | 4.0^†^ |
| Trap position | 1 | 24 | 12.3** |
| Age X position | 1 | 24 | 2.4 |
| Richness X position | 1 | 24 | 0.6 |
| **Variance components** | **Var.** | **SE** |  |
| Exposure period | -0.05 | 0.04 |  |
| Plot | 0.13 | 0.07 |  |
| Residual variance | 0.11 | 0.03 |  |
| **B** |  |  |  |
| Altitude | 1 | 23 | 0.0 |
| Stand age | 1 | 23 | 0.3 |
| Tree richness | 1 | 21.4 | 1.0 |
| Trap position | 1 | 24 | 7.8* |
| Age X position | 1 | 24 | 1.8 |
| Richness X position | 1 | 24 | 0.2 |
| **Variance components** | **Var.** | **SE** |  |
| Exposure period | 0.02 | 0.15 |  |
| Plot | 0.17 | 0.16 |  |
| Residual variance | 0.18 | 0.05 |  |
| **C** |  |  |  |
| Altitude | 1 | 22.9 | 0.0 |
| Stand age | 1 | 23 | 6.4* |
| Tree richness | 1 | 22.5 | 2.9 |
| Trap position | 1 | 24 | 129.1*** |
| Age X position | 1 | 24 | 7.3* |
| Richness X position | 1 | 24 | 3.4^†^ |
| **Variance components** | **Var.** | **SE** |  |
| Exposure period | 0.00 | 0.62 |  |
| Plot | 1.03 | 0.76 |  |
| Residual variance | 0.86 | 0.25 |  |
| **D** |  |  |  |
| Altitude | 1 | 23 | 1.8 |
| Stand age | 1 | 23 | 0.1 |
| Tree richness | 1 | 21.7 | 1.8 |
| Trap position | 1 | 24 | 0.0 |
| Age X position | 1 | 24 | 0.3 |
| Richness X position | 1 | 24 | 0.5 |
| **Variance components** | **Var.** | **SE** |  |
| Exposure period | 0.02 | 0.12 |  |
| Plot | 0.15 | 0.13 |  |
| Residual variance | 0.13 | 0.04 |  |

d.f., degrees of freedom; denominator d.f., denominator degrees of freedom,

F, conditional F-statistic; Var., variance component estimate and SE, standard errors for random effects; ^†^P<0.1, *****P < 0.05, ******P < 0.01, *******P < 0.001

**
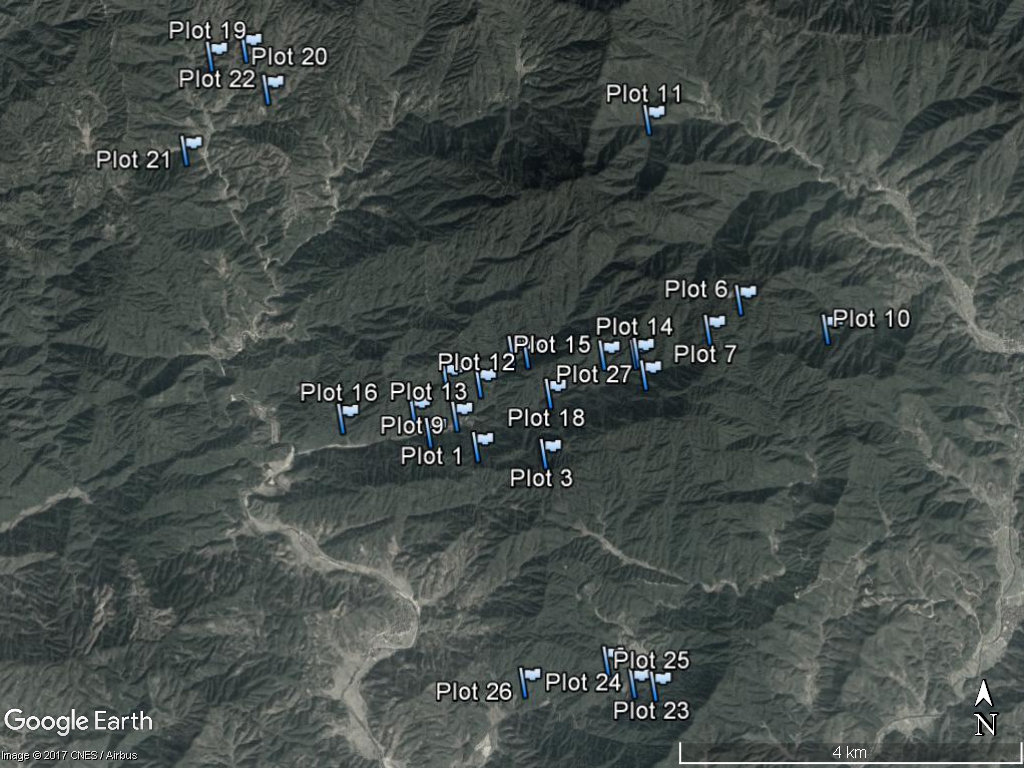
**

**Fig. S1 The distribution of plots on the landscape.** The area covers approximately 38 km^2^ extending 8 km north to south and 6.5 km east to west.


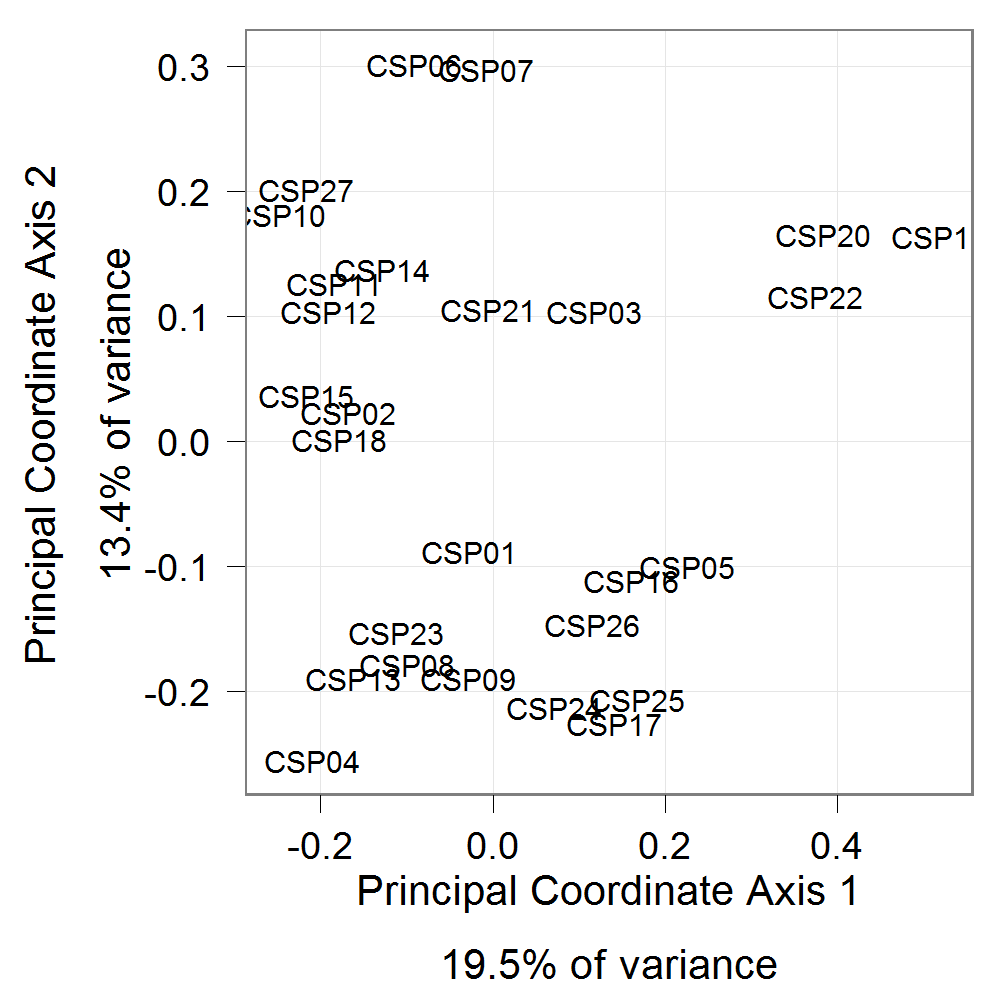


**Fig. S2** **The PCoA of tree species presence-absence data and the Jaccard dissimilarity index.** The axes of this analysis were used as predictor variables for the constrained analysis of proximities and as linear predictors of arthropod PCoA axes. Numbers are the plot identifiers.

**
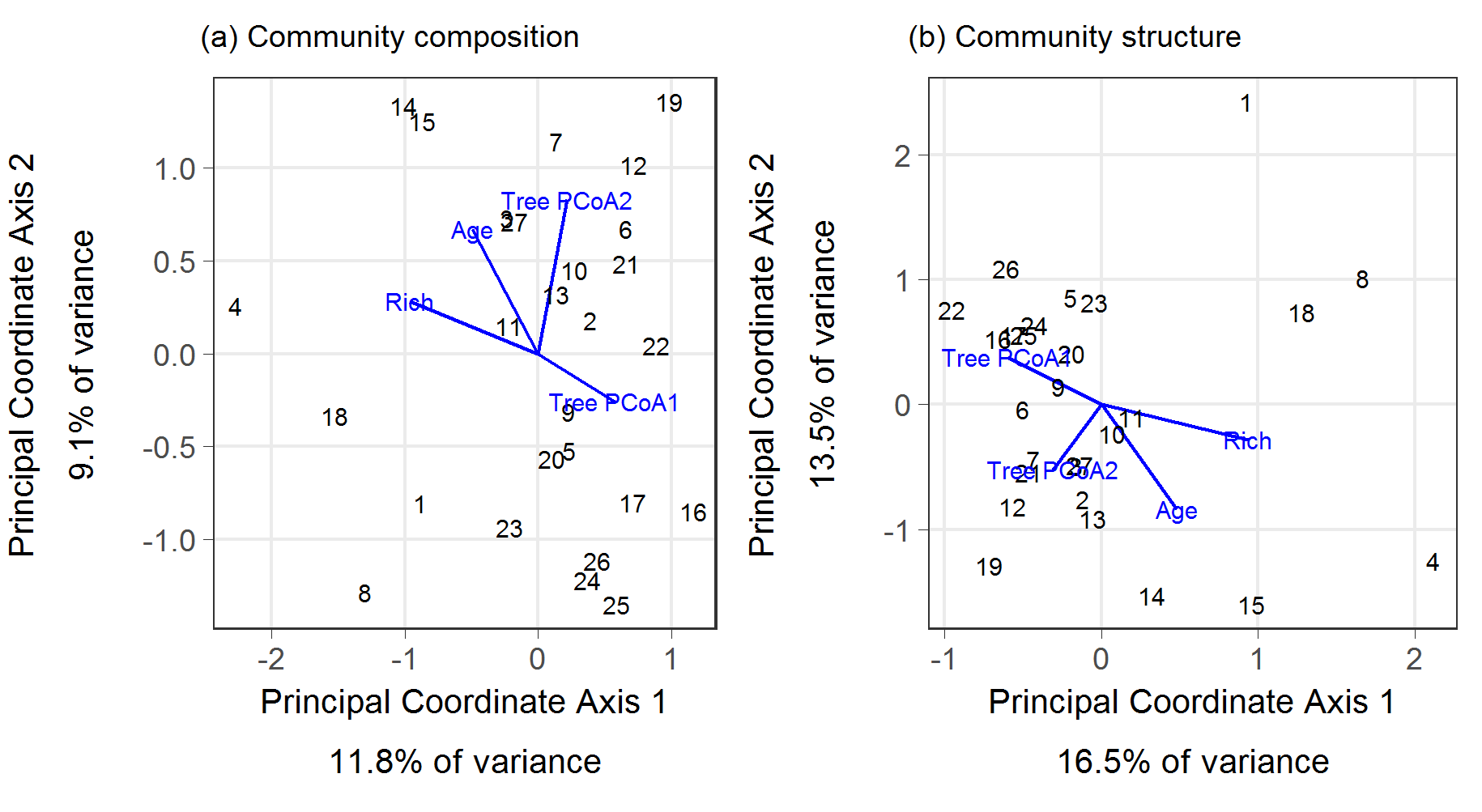
**

**Fig. S3** Results of the distance-based redundancy analyses on the (a) Jaccard and (b) Bray-Curtis dissimilarity matrices of the predator community. The constraining variable of rich marginally explained variation in the PCoA of predator composition (P < 0.1). Numbers are the plot identifiers.

**
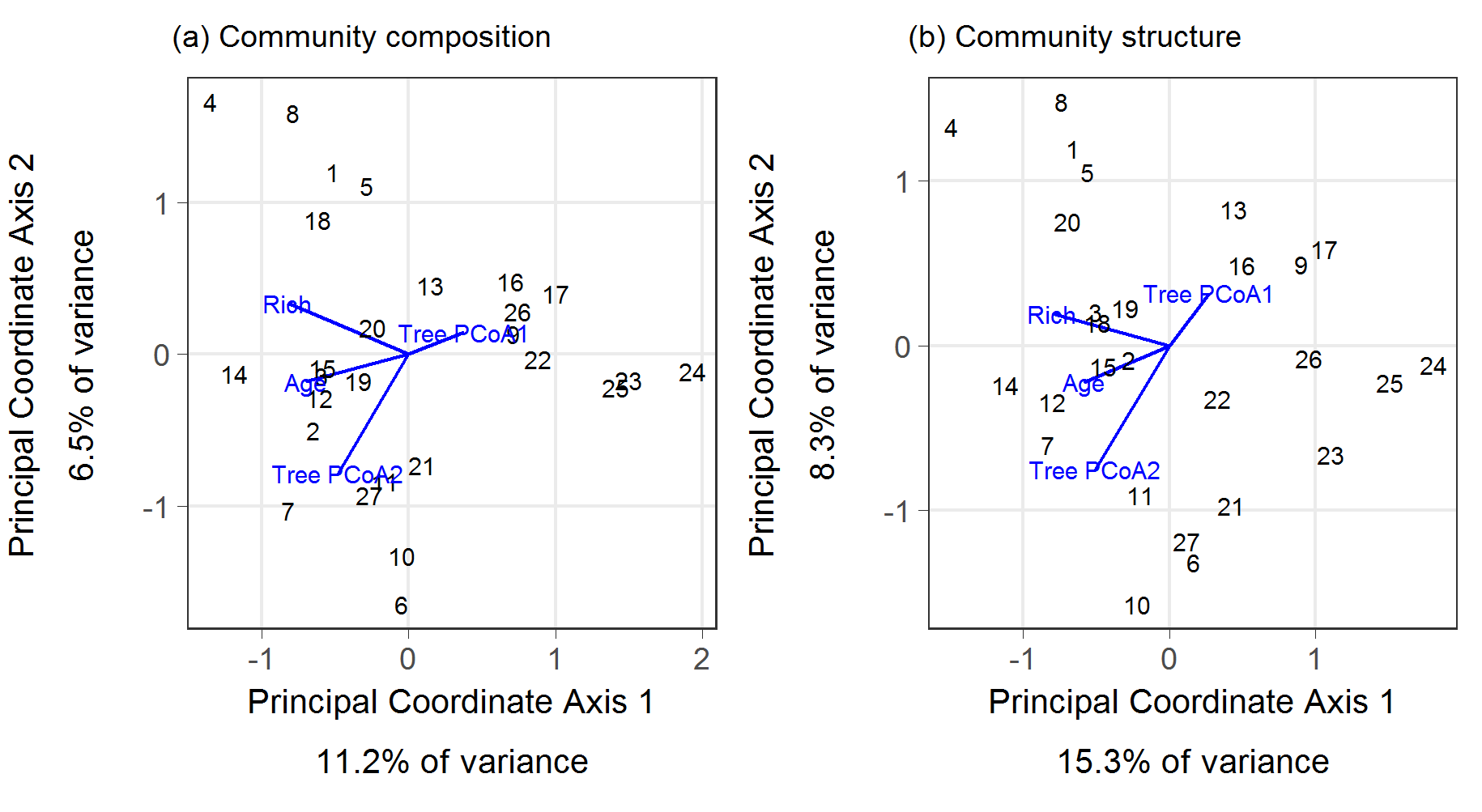
**

**Fig. S4** Results of the distance-based redundancy analyses on the (a) Jaccard and (b) Bray-Curtis dissimilarity matrices of the herbivore community. The constraining variable of PCoA axis 2 of tree composition marginally explained variation in the PCoA of herbivore composition (P < 0.1). Numbers are the plot identifiers.

**
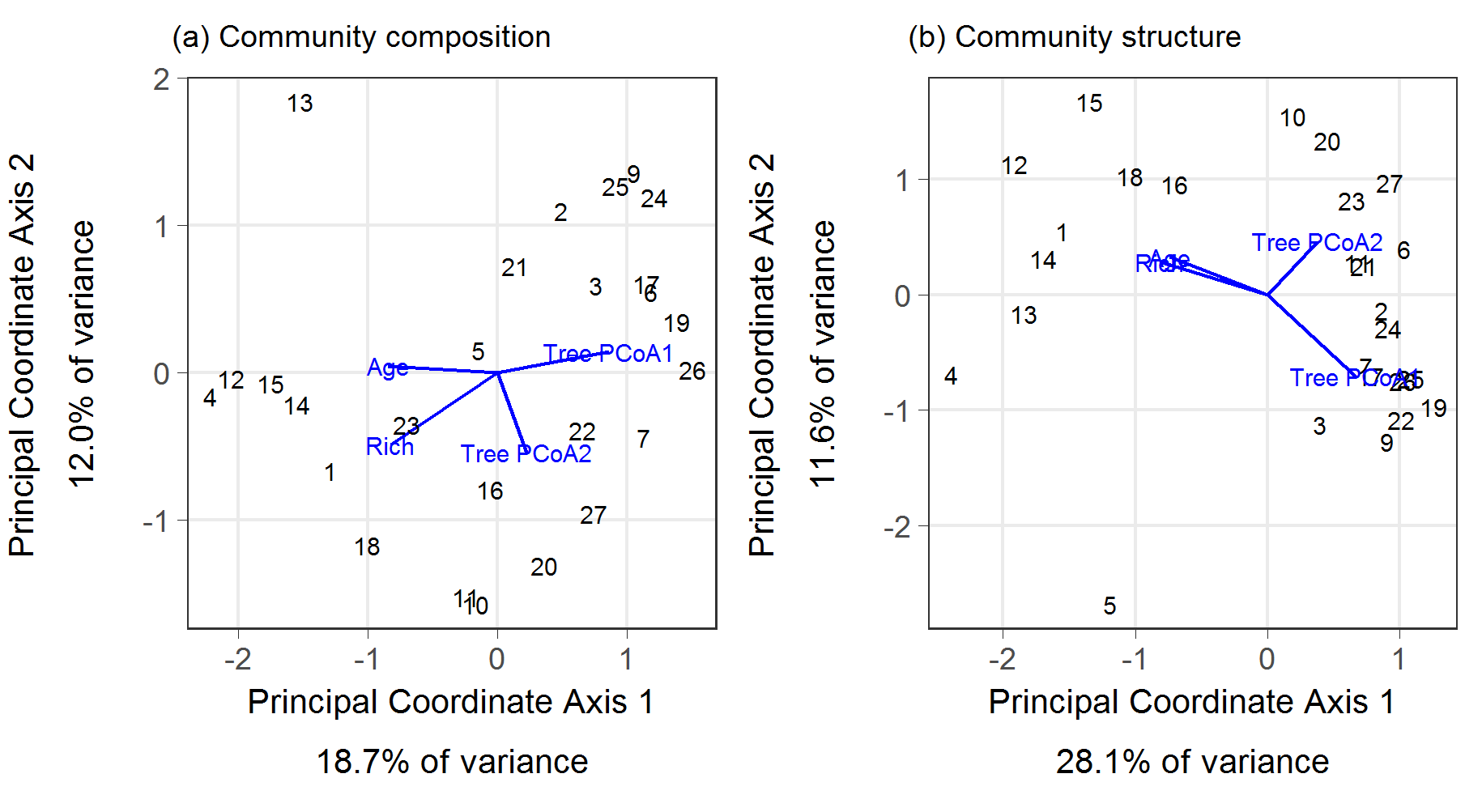
**

**Fig. S5** Results of the distance-based redundancy analyses on the (a) Jaccard and (b) Bray-Curtis dissimilarity matrices of the detritivore community. The constraining variable of PCoA axis 1 of the tree community marginally explained variation in the PCoA of detritivore composition (P < 0.1). Numbers are the plot identifiers.

**
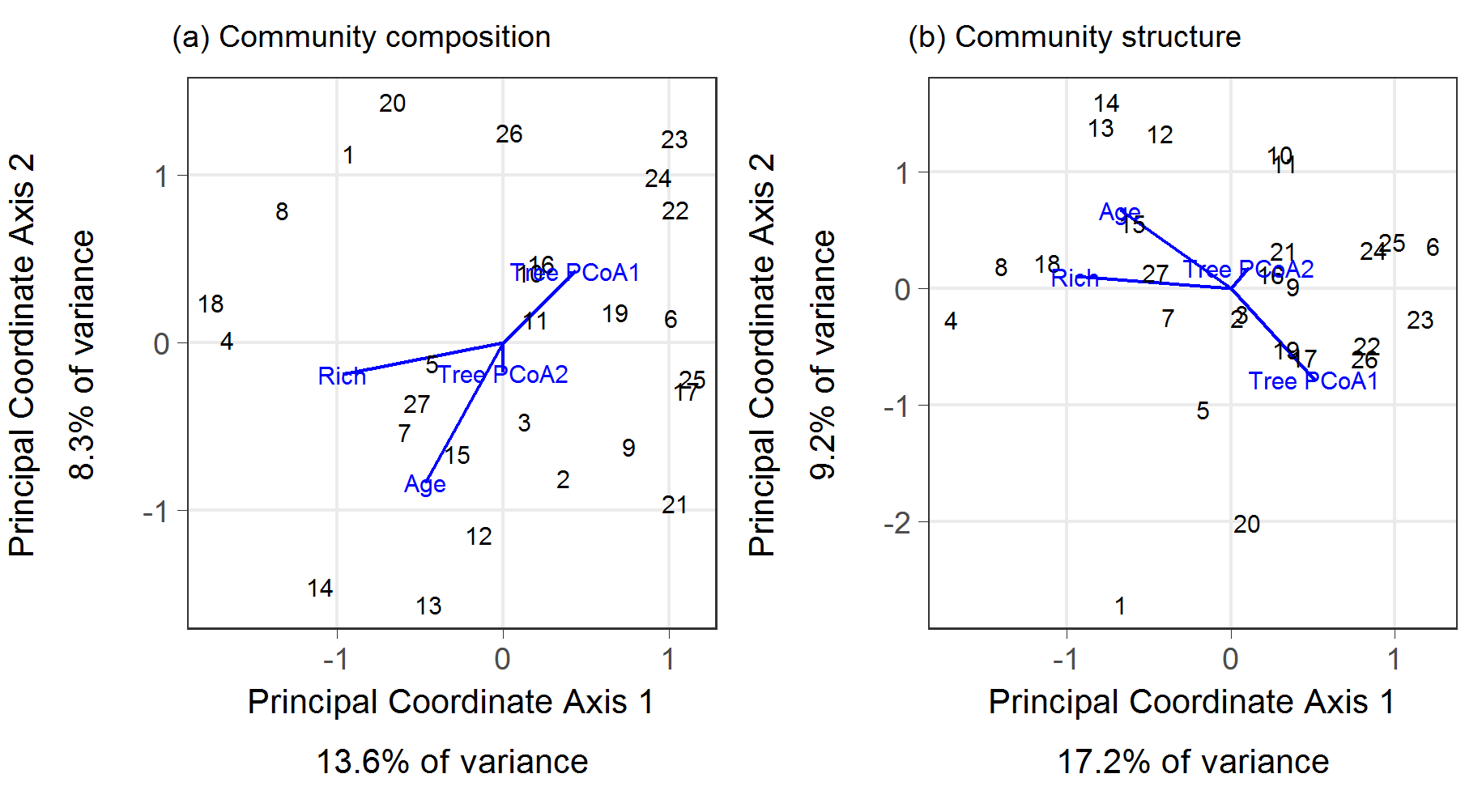
**

**Fig. S6** Results of the distance-based redundancy analyses on the (a) Jaccard and (b) Bray-Curtis dissimilarity matrices of the pollinator community. The constraining variable of rich marginally explained variation in the PCoA of pollinator composition (P < 0.1). Numbers are the plot identifiers.

**
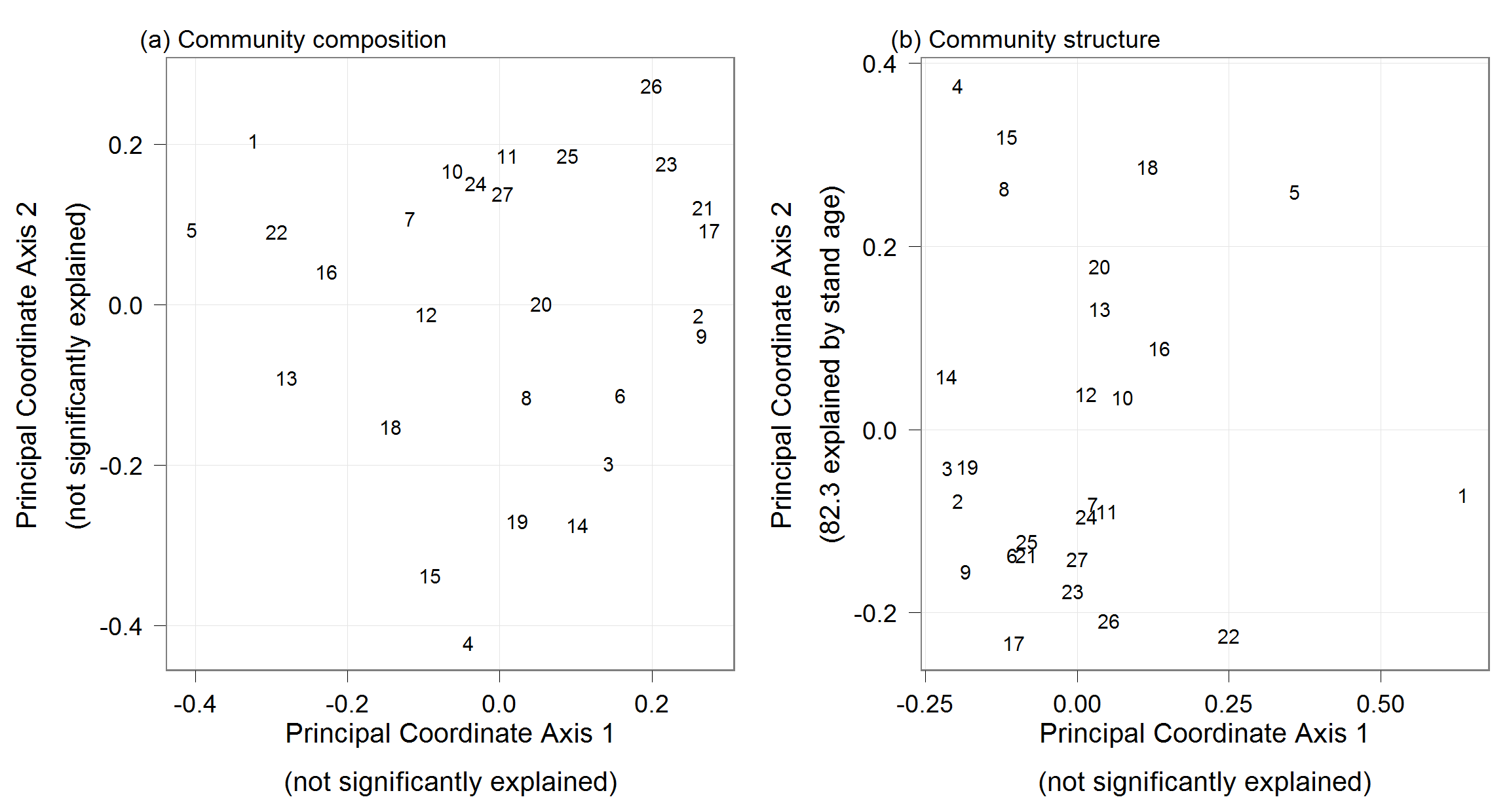
**

**Fig. S7** (a) PCoA of the predator community composition based on the Jaccard dissimilarity index. Neither axis was poorly explained by any variables. (b) PCoA of the predator community structure based on the Bray-Curtis dissimilarity index. Axis one was poorly explained by all variables while axis two was explained by stand age. Therefore, predator community was independent of tree composition. Numbers are the plot identifiers.

**
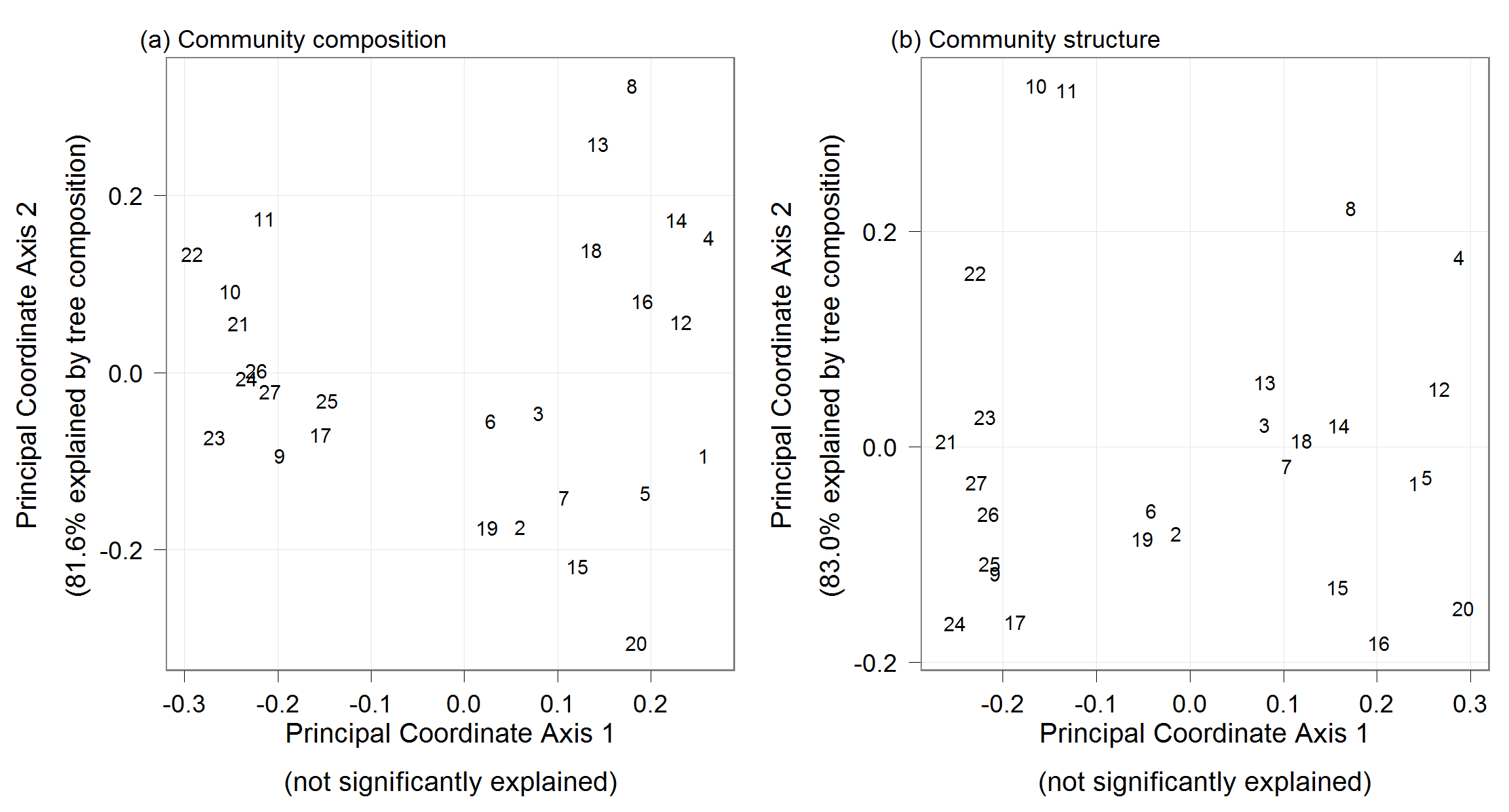
**

**Fig. S8** (a) PCoA of the herbivore community composition based on the Jaccard dissimilarity index. Axis two was explained by the PCoA axis one of tree composition. (b) PCoA of the herbivore community structure based on the Bray-Curtis dissimilarity index. Axis one was poorly explained by all variables while axis two was explained by axis one of the PCoA of tree composition. Therefore, herbivore community composition and structure was partially affected by tree composition. Numbers are the plot identifiers.

**
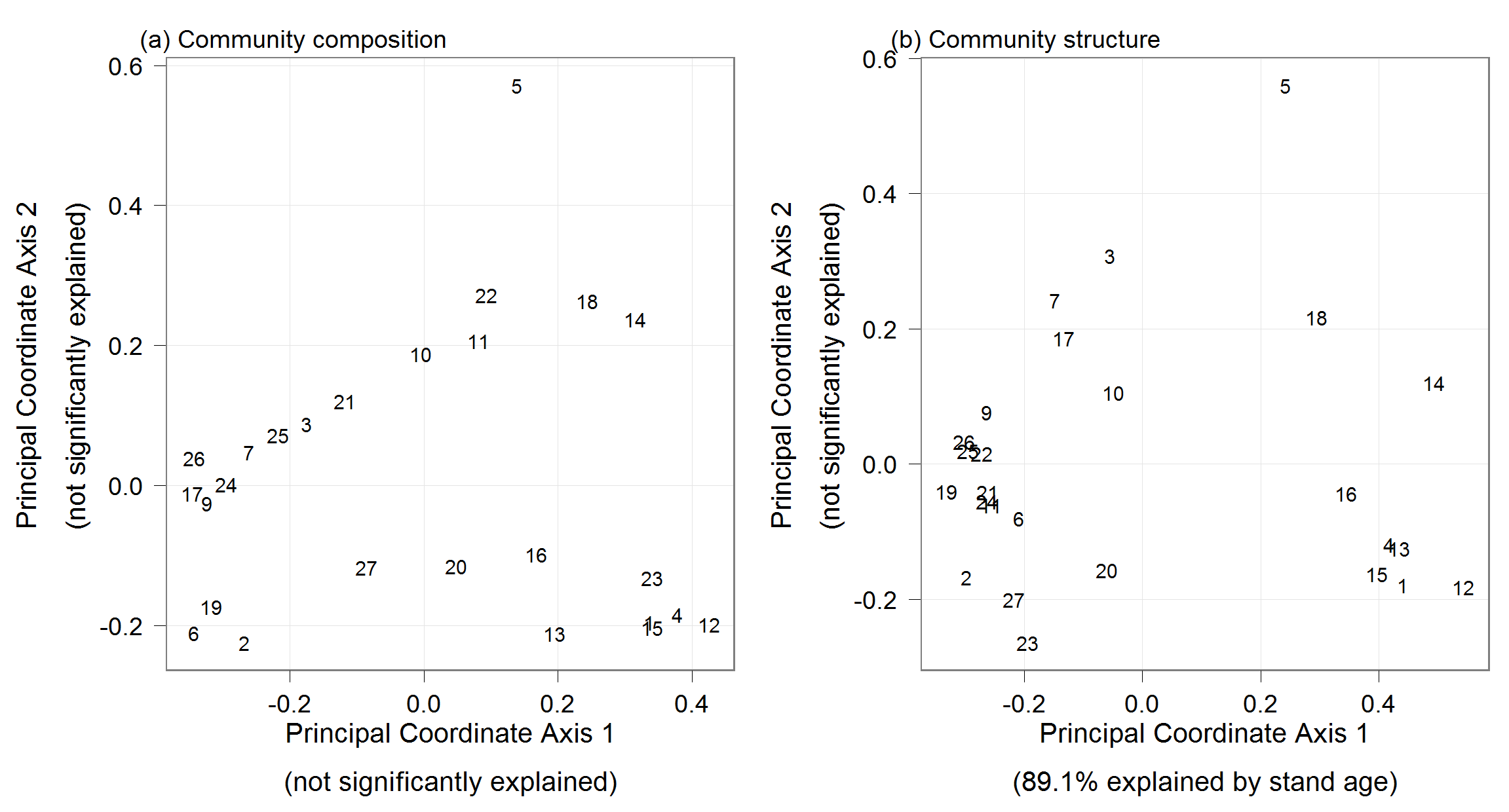
**

**Fig. S9** (a) PCoA of the detritivore community composition based on the Jaccard dissimilarity index. Neither axis was significantly explained by any variables. (b) PCoA of the herbivore community structure based on the Bray-Curtis dissimilarity index. Axis one was explained by stand age. Therefore, detritivore community composition and structure was independent of tree composition. Numbers are the plot identifiers.

**
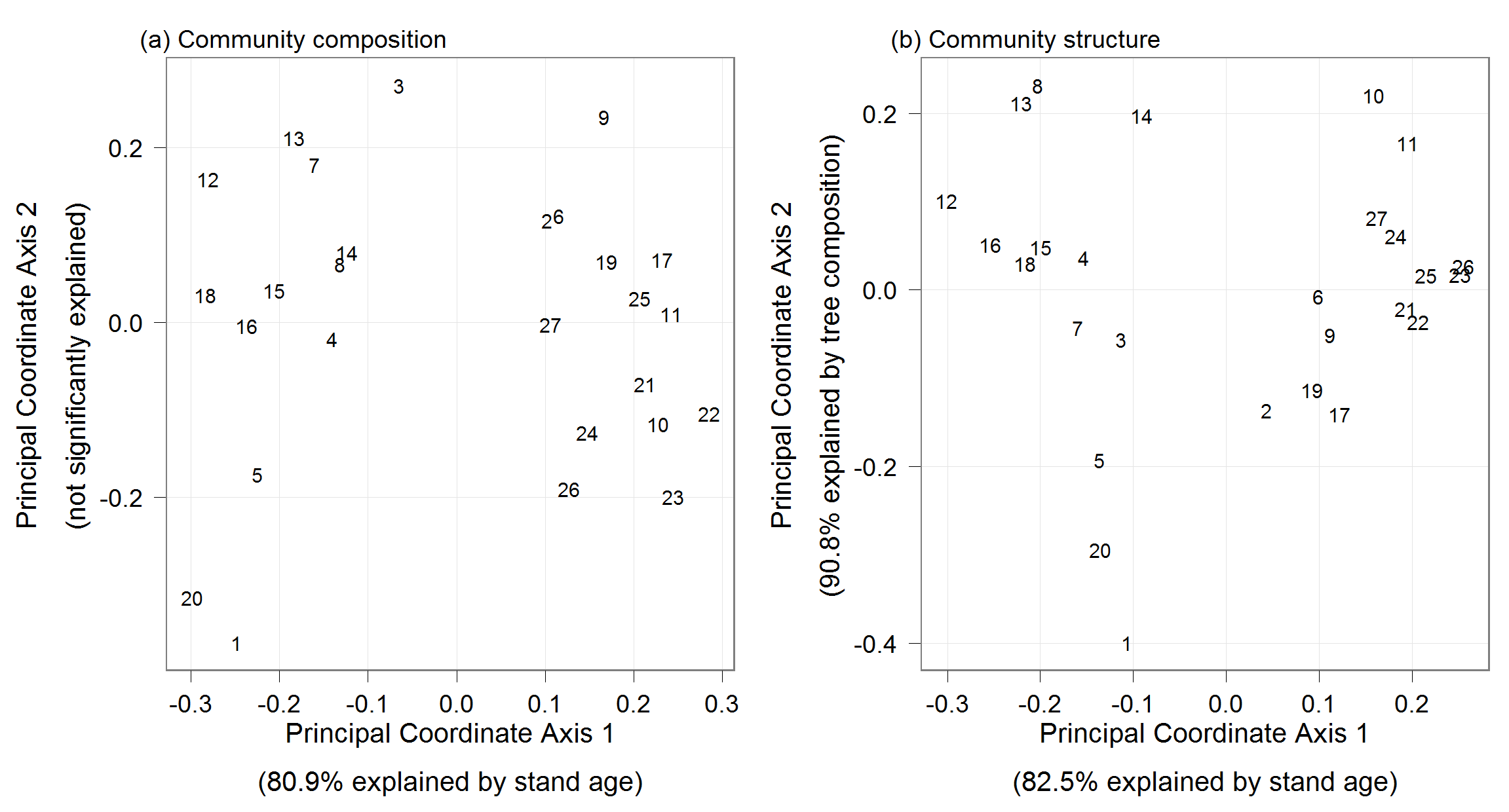
**

**Fig. S10** (a) PCoA of the pollinator community composition based on the Jaccard dissimilarity index. Axis one was explained by stand age. (b) PCoA of the pollinator community structure based on the Bray-Curtis dissimilarity index. Axis one was explained by stand age while axis two was explained by axis one of the PCoA of tree composition. Therefore, pollinator community structure was partially affected by tree composition. Numbers are the plot identifiers.
